# Drug Survival and Predictors of Systemic Treatment Outcome in Atopic Dermatitis: Data From a Nationwide Swedish Cohort

**DOI:** 10.2340/actadv.v105.43464

**Published:** 2025-08-07

**Authors:** Pontus O. JONSSON, Mahsa TAYEFI, Axel SVEDBOM, Maria BRADLEY, Emma K. JOHANSSON

**Affiliations:** 1 Dermatology and Venereology Unit, Department of Medicine Solna, Karolinska Institutet, Stockholm, Sweden; 2 Department of Dermatology, Karolinska University Hospital, Stockholm, Sweden

**Keywords:** atopic dermatitis, dupilumab, Janus kinase inhibitors, survival analysis

## Abstract

Real-world data on the drug survival of established and emerging treatment options in atopic dermatitis provide a comprehensive measure of the efficacy and tolerability of these interventions, which may enable improvements in clinical management. This study aimed to describe the drug survival, with associated predictors, of treatment with abrocitinib, baricitinib, cyclosporine, dupilumab, methotrexate, tralokinumab, and upadacitinib among patients with atopic dermatitis in Sweden who were recruited into the multicentre prospective SwedAD cohort between January 2017 and April 2024. A total of 1,194 patients were included with a total of 1,486 treatment episodes. The 2-year drug survival probability was 14.2% for baricitinib (treatment episodes, *n* = 30), 12.2% for cyclosporine (*n* = 40), 79.7% for dupilumab (*n* = 1,026), 45.4% for methotrexate (*n* = 260), and 46.0% for upadacitinib (*n* = 89). Two-year follow-up data were not available for abrocitinib (*n* = 23) or tralokinumab (*n* = 18). All drugs were compared with the most used conventional systemic treatment at study baseline, methotrexate, using Cox regression. Only dupilumab showed a significantly lower hazard rate of drug discontinuation. In conclusion, dupilumab therapy demonstrated longer drug survival than methotrexate in atopic dermatitis. The impact of national treatment guidelines and time since drug approval should be considered when interpreting the results.

## SIGNIFICANCE

Using data from SwedAD, a nationwide Swedish quality registry for patients with atopic dermatitis, our analysis of 1,486 episodes of systemic medical treatment in 1,194 patients between the years 2017 and 2024 showed that those receiving the biological drug dupilumab were less likely to discontinue treatment, for any reason, than those receiving methotrexate, the traditionally most used treatment in Sweden. Dupilumab also performed well compared with other investigated treatment options. After 2 years of treatment, 79.7% of patients were still using dupilumab, compared with 45.4% for methotrexate, indicating greater effect and a more tolerable side effect profile.

Atopic eczema, also known as atopic dermatitis (AD), is a chronic skin disease characterized by dry skin and inflamed, pruritic skin lesions due to a type-2-skewed immune response (1). The condition has a profound impact on the disease-related quality of life of patients and their families (2). With a prevalence of up to 17% in adults and 22% in children, AD is the most common inflammatory skin disease, and a major cause of disease burden globally (3, 4). The annual societal financial impact of AD in Europe is estimated at €30 billion, roughly half of which is indirect costs attributed to reduced worker performance and difficulties for patients to access the job market (5).

Considering the prevalence and impact of AD, significant efforts have been invested in the development of novel therapeutic agents targeting the key pathogenic processes in this condition. New treatment options approved for use in moderate to severe AD, including the fully human monoclonal antibody dupilumab and small-molecule inhibitors of the Janus kinase (JAK) enzyme pathway, have shown improved treatment efficacy compared with established AD treatment regimens with conventional immunomodulators such as cyclosporine A (CsA) and methotrexate (MTX) (6, 7). Dupilumab inhibits interleukin 4 (IL-4) and IL-13 signalling – both important mediators of the type-2 immune response – by targeting the shared IL-4α subunit (8). IL-13 signalling is also the target of tralokinumab, a human monoclonal IL-13 antibody approved for use in moderate to severe AD (9, 10). To facilitate a rational, safe, and effective use of systemic treatment in AD, at both the individual and the societal level, we aimed to investigate real-world treatment outcomes of emerging and established treatment options in this condition. Drug survival, a survival analysis of the event “drug discontinuation”, summarizes the efficacy and tolerability of a treatment and is a comprehensive measure of a drug’s utility in a clinical setting (11).

Our aim was to investigate the 2-year drug survival rate for treatment with CsA, dupilumab, JAK inhibitors (abrocitinib, baricitinib, upadacitinib), MTX, and tralokinumab in Swedish AD patients, and to describe potential predictors of drug survival and reasons for drug discontinuation.

## MATERIALS AND METHODS

All patients registered in SwedAD, a multicentre prospective registry of AD patients, both children and adults, receiving systemic treatment at 51 dermatological clinics in Sweden, were eligible for inclusion in the study (12). SwedAD collects data on treatment, patient-reported outcome measures, and clinical variables. Treatment episodes (initiation of systemic treatment) registered between 1 January 2017 and 1 March 2024 were considered for inclusion in the analysis. Only drugs with at least 10 registered treatment episodes were included. Treatment episodes with >1 concurrent substances and patients with missing data (no visit reported within the first 12 months after treatment initiation) were excluded from the analysis. Only the first treatment episode with each drug per patient was included, and treatment interruptions of more than 90 days were considered discontinuations. The SwedAD register does not mandate precise information regarding systemic treatment initiated prior to inclusion in the registry and therefore prior use of systemic treatment was not included as a variable in the analysis.

### Covariates

The following demographic and clinical information was recorded at baseline: sex, age, weight, age at onset, age at initiation of systemic treatment, presence of atopic comorbidities, education level, smoking status, alcohol use (≥2 times weekly). The analysed covariates represent a subset of the variables recorded in the SwedAD registry. Weight, smoking status, and alcohol use are reassessed annually. At baseline and at subsequent visits, eczema severity is graded using the Eczema Area and Severity Index (EASI). At each visit, treatment status is registered, along with – in applicable cases – the reason for discontinuation: “other cause”, “side effects”, “lack of efficacy”, “disease remission”, or “temporary discontinuation”. A decision to discontinue treatment is made jointly by the physician and patient. Patient-reported outcome measures are collected at each visit: Itch Numeric Rating Scale (itch-NRS), Patient-Oriented Eczema Measure (POEM), Montgomery-Åsberg Depression Rating Scale-Self-report (MADRS-S), and Dermatology Life Quality Index (DLQI). Further, any immunosuppressive therapy initiated since enrolment in SwedAD is registered. SwedAD does not entail a fixed visit schedule, but a baseline visit and annual re-visits are standard; however, precise visit schedule varies between participating clinics.

### Statistical analysis

Descriptive analysis of the demographic and clinical variables was performed using absolute numbers and percentages for categorical variables and medians and interquartile ranges (IQRs) for numerical variables. Overall drug survival, discontinuation due to ineffectiveness, and discontinuation due to adverse events were analysed with Kaplan–Meier survival curves for each treatment type. Patients were censored if treatment was ongoing at data lock or if lost to follow-up (>15 months since last visit). The drug survival for each treatment was compared with MTX as reference using the Cox proportional hazard model.

The following variables were analysed as potential predictors of overall drug survival: age at treatment start, sex, weight, smoking status, education level, baseline level of EASI, POEM, DLQI, and itch-NRS. For patients who remained on treatment at 6 months, EASI, POEM, DLQI, itch-NRS, and MADRS-S scores, as well as the achievement of minimal clinically important differences for EASI (6.6 points), DLQI (4.0 points), POEM (3.4 points), MADRS-S (3–6 points), and itch-NRS (≥ 2–4 points), were included in the analysis (13–16).

Univariable and multivariable Cox regression analysis was performed for each treatment (the JAK inhibitors were analysed as a combined treatment group). Predictors with a statistically significant effect on drug survival in the univariable analysis were adjusted in a multivariable analysis to confirm the effect. A *p*-value of < 0.05 was considered significant. Multiple imputation and complete case analysis were used for the multivariable and univariable Cox regressions, respectively. Statistical analyses were performed using statistical software (Stata, version 17.2, StataCorp LLP, College Station, TX, USA).

### Ethics

The study was approved by the Swedish Ethical Review Authority (reference number: 2010/345-31/2, 2022-01853-01, 2023-06612-02).

## RESULTS

A total of 1,486 treatment episodes in 1,194 patients fulfilled the inclusion criteria. Paediatric patients (0 to 17 years of age) accounted for 10.6% of the treatment episodes. Median patient age [IQR] at treatment start was 35 years [24, 53], ranging between 3 and 88 years, 608 (50.9%) were female and the median EASI score at baseline was 13 [6, 21]. Dupilumab was the most common treatment type and accounted for 1,026 treatment episodes, followed by MTX (*n* = 260) and, in decreasing order, upadacitinib (*n* = 89), CsA (*n* = 40), baricitinib (*n* = 30), abrocitinib (*n* = 23), and tralokinumab (*n* = 18). Baseline EASI scores were higher among patients treated with CsA (**Table I**).

**Table I:** Patient baseline and background characteristics, per treatment

Item	Abrocitinib *n* = 23	Baricitinib *n* = 30	Cyclosporine A *n* = 40	Dupilumab *n* = 1,026	Methotrexate *n* = 260	Tralokinumab *n* = 18	Upadacitinib *n* = 89
Male sex, *n* (%)	13 (56.5)	15 (50.0)	18 (45.0)	504 (49.1)	123 (47.3)	7 (38.9)	45 (50.6)
Age at treatment start, median (IQR)	32 (28, 53)	28 (25, 41)	29 (21, 35)	35 (24, 54)	35 (24, 53)	36 (30, 59)	30 (23, 42)
Current smoker, *n* (%)	2/13 (15.4)	2/24 (8.3)	5/27 (18.5)	72/561 (12.8)	28/195 (14.4)	1/13 (7.7)	4/58 (6.9)
Missing data, *n*	10	6	13	465	65	5	31
Atopic comorbidities*, *n* (%)	13/14 (92.9)	20/23 (87.0)	22/29 (75.9)	460/542 (75.9)	163/205 (79.5)	7/10 (70.0)	48/53 (90.6)
Missing data, *n*	9	7	11	484	55	8	36
Age at onset of AD, *n* (%)							
< 2 years	6/12 (50.0)	14/22 (63.6)	8/28 (28.6)	281/535 (52.5)	106/198 (53.5)	5/10 (50.0)	33/50 (66.0)
2–19 years	4/12 (33.3)	7/22 (31.8)	15/28 (53.6)	199/535 (37.2)	77/198 (38.9)	3/10 (30.0)	16/50 (32.0)
≥ 20 years	2/12 (16.7)	1/22 (4.5)	5/28 (17.9)	55/535 (10.3)	15/198 (7.6)	2/10 (20.0)	1/50 (2.0)
Missing data, *n*	11	8	12	491	62	8	39
Highest level of education, *n* (%)							
Primary or secondary school	5/12 (41.7)	4/20 (20.0)	11/27 (40.7)	221/495 (44.6)	95/193 (49.2)	4/8 (50.0)	18/46 (39.1)
University	5/12 (41.7)	10/20 (50.0)	14/27 (51.9)	228/495 (46.1)	84/193 (43.5)	4/8 (50.0)	24/46 (52.2)
Other	2/12 (16.7)	6/20 (30.0)	2/27 (7.4)	46/495 (9.3)	14/193 (7.3)	0/8 (0.0)	4/46 (8.7)
Missing data, *n*	11	10	13	531	67	10	43
Alcohol use**, *n* (%)	1/7 (14.3)	0/14 (0.0)	0/8 (0.0)	16/368 (4.3)	8/124 (6.5)	0/7 (0.0)	1/35 (2.9)
Missing data, *n* (%)	16 (69.6)	16 (53.3)	32 (80.0)	658 (64.1)	136 (52.3)	11 (61.1)	54 (60.7)
Weight, kilograms, median (IQR)	71.0 (65.0, 99.0) (*n* = 10)	65.0 (63.2, 75.0) (*n* = 25)	70.0 (50.4, 86.5) (*n* = 31)	73.0 (60.0, 85.0) (*n* = 557)	73.0 (63.3, 84.5) (*n* = 170)	70.8 (65.0, 85.0) (*n* = 10)	71.0 (63.0, 86.0) (*n* = 53)
Missing data, *n*	13	5	9	469	90	8	36
EASI at baseline, median (IQR)	8 (5, 15) (*n* = 18)	10 (5, 12) (*n* = 16)	20 (11, 26) (*n* = 21)	13 (6, 22) (*n* = 814)	11 (5, 19) (*n* = 176)	10 (7, 23) (*n* = 15)	9 (4, 19) (*n* = 67)
EASI at 6 months***, median (IQR)	4 (1, 8) (*n* = 8)	7 (4, 13) (*n* = 13)	8 (4, 17) (*n* = 15)	2 (1, 5) (*n* = 468)	4 (1, 6) (*n* = 97)	7 (3, 15) (*n* = 4)	2 (1, 7) (*n* = 41)
DLQI at baseline, median (IQR)	12 (10, 14) (*n* = 17)	12 (9, 18) (*n* = 17)	18 (11, 23) (*n* = 16)	15 (9, 20) (*n* = 700)	13 (8, 18) (*n* = 157)	14 (9, 18) (*n* = 13)	13 (7, 19) (*n* = 54)
DLQI at 6 months***, median (IQR)	8 (1, 11) (*n* = 7)	12 (9, 22) (*n* = 15)	9 (5, 15) (*n* = 11)	3 (1, 7) (*n* = 413)	6 (2, 13) (*n* = 96)	18 (2, 20) (*n* = 3)	6 (2, 11) (*n* = 39)
POEM at baseline, median (IQR)	12 (9, 14) (*n* = 11)	10 (6, 16) (*n* = 11)	13 (10, 22) (*n* = 13)	13 (7, 20) (*n* = 507)	12 (6, 17) (*n* = 78)	15 (5, 26) (*n* = 12)	10 (6, 18) (*n* = 45)
POEM at 6 months***, median (IQR)	7 (2, 11) (*n* = 4)	10 (6, 16) (*n* = 8)	15 (4, 22) (*n* = 7)	6 (2, 12) (*n* = 312)	9 (5, 14) (*n* = 52)	6 (4, 22) (*n* = 3)	7 (4, 12) (*n* = 33)
MADRS-S at baseline, median (IQR)	12 (9, 14) (*n* = 11)	10 (6, 16) (*n* = 11)	13 (10, 22) (*n* = 13)	13 (7, 20) (*n* = 507)	12 (6, 17) (*n* = 78)	15 (5, 26) (*n* = 12)	10 (6, 18) (*n* = 45)
MADRS-S at 6 months***, median (IQR)	7 (2, 11) (*n* = 4)	12 (6, 19) (*n* = 9)	15 (4, 22) (*n* = 7)	6 (2, 11) (*n* = 318)	9 (5, 13) (*n* = 53)	6 (4, 22) (*n* = 3)	7 (4, 12) (*n* = 33)
Itch-NRS at baseline, median (IQR)	7 (4, 8) (*n* = 15)	7 (5, 8) (*n* = 17)	8 (4, 9) (*n* = 16)	7 (5, 8) (*n* = 685)	6 (4, 8) (*n* = 130)	7 (5, 8) (*n* = 13)	6 (4, 7) (*n* = 55)
Itch-NRS at 6 months***, median (IQR)	3 (1, 6) (*n* = 7)	6 (5, 7) (*n* = 11)	6 (5, 8) (*n* = 11)	2 (1, 4) (*n* = 417)	3 (2, 6) (*n* = 93)	3 (2, 7) (*n* = 3)	3 (2, 6) (*n* = 38)

IQR: interquartile range; AD: atopic dermatitis; EASI: Eczema Area and Severity Index; DLQI: Dermatology Life Quality Index; POEM: Patient-Oriented Eczema Measure; MADRS-S: Montgomery-Åsberg Depression Rating Scale-Self-report; Itch-NRS: Itch Numeric Rating Scale.

* Asthma, allergic rhinoconjunctivitis,

** alcohol use ≥2 times weekly,

*** aAs measured on the visit closest to 6 months (within 3 to 9 months) after treatment initiation.

**Fig. 1:**
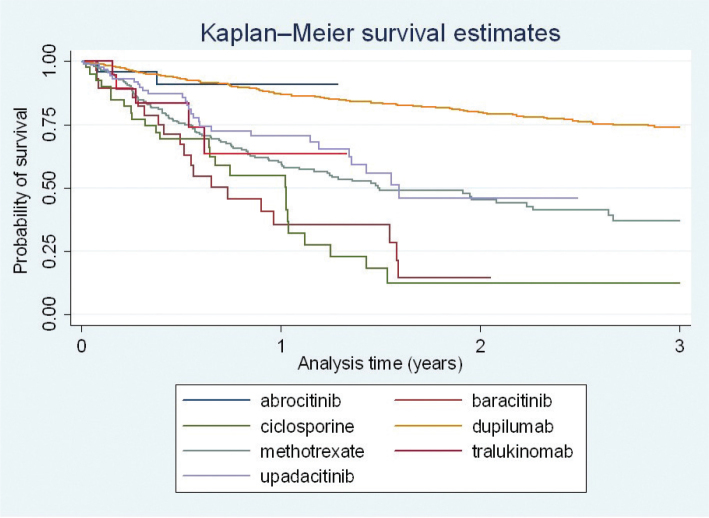
Drug survival, all treatments.

The 2-year overall drug survival rate of dupilumab was 79.7% and the corresponding survival rates for the other treatment types were 14.2% for baricitinib, 12.2% for CsA, 45.4% for MTX, and 46.0% for upadacitinib (**Fig. 1**). Data on overall drug survival at 2 years were not available for abrocitinib or tralokinumab. The 1-year overall drug survival for each treatment type was, in decreasing order, 90.9% for abrocitinib, 86.9% for dupilumab, 70.4% for upadacitinib, 63.5% for tralokinumab, 60.0% for MTX, 55.0% for CsA, and 35.6% for baricitinib.

The Cox proportional hazard model showed that dupilumab had a significant lower rate of drug discontinuation compared with MTX (hazard ratio (HR) 0.27, 95% confidence interval (CI) 0.20–0.34), *p* < 0.01) (**Table II**). Baricitinib and CsA showed a significantly higher rate of drug discontinuation compared with MTX, with an HR of 1.96 (95% CI 1.27–3.03, *p* < 0.01) for baricitinib and 1.70 (95% CI 1.13–2.54, *p* < 0.01) for CsA, respectively.

**Table II:** Rate of drug discontinuation compared with methotrexate, measured using the Cox proportional hazard model

Treatment	HR	SD	P >|z|	95% CI
Abrocitinib	0.28	0.20	0.08	0.07–1.17
Baricitinib	1.96	0.44	< 0.01	1.27–3.03
Dupilumab	0.27	0.04	< 0.01	0.20–0.34
Tralokinumab	0.99	0.45	0.99	0.41–2.40
Cyclosporine	1.70	0.35	0.01	1.13–2.54
Upadacitinib	0.84	0.18	0.43	0.56–1.28

HR: hazard ratio; SD: standard deviation; CI: confidence interval.

In total, 405 (27.3%) out of 1,486 treatment episodes were discontinued. Lack of efficacy was the leading cause, accounting for 150 treatment cessations (37%), followed by side effects (29%), other causes (22%), remission (8%), and temporary discontinuation (3%) (**Table III**). Detailed description of the exact reason for treatment discontinuation was not available in all cases. Infections and injection-related discomfort were among the most commonly reported reasons for treatment cessation due to “side effects”, while discontinuations due to “other causes” reflected a range of individual, often patient-specific circumstances such as participation in clinical trials, comorbid conditions, fear of injection or injection fatigue and, unfortunately, difficulties in access to the prescribed medication. At data lock, 17.4% of treatment episodes with abrocitinib had been discontinued, whereas the corresponding proportions for the other drugs were, in increasing order, dupilumab 18.2%, tralokinumab 27.8%, upadacitinib 39.3%, MTX 48.1%, CsA 67.5% and baricitinib 73.3%. In patients receiving dupilumab, discontinuations were due to lack of efficacy (28.3%), other cause (27.8%), side effects (27.3%), disease remission (12.3%), or temporary discontinuation (4.3%).

**Table III:** Cause of treatment discontinuation

Treatment	Cause of treatment discontinuation					Total
	Other cause	Side effects	Lack of efficacy	Disease remission	Temporary discontinuation	
Abrocitinib % (*n*)	0	50.0 (2)	50.0 (2)	0	0	17.4 (4/23)
Baricitinib % (*n*)	13.6 (3)	13.6 (3)	63.6 (14)	0	9.1 (2)	73.3 (22/30)
Cyclosporine % (*n*)	44.4 (12)	3.7 (1)	40.7 (11)	7.4 (2)	3.7 (1)	67.5 (27/40)
Dupilumab % (*n*)	27.8 (52)	27.3 (51)	28.3 (53)	12.3 (23)	4.3 (8)	18.2 (187/1,026)
Methotrexate % (*n*)	15.2 (19)	34.4 (43)	40.8 (51)	7.2 (9)	2.4 (3)	48.1 (125/260)
Tralokinumab % (*n*)	0	40.0 (2)	60.0 (3)	0	0	27.8 (5/18)
Upadacitinib % (*n*)	11.4 (4)	42.9 (15)	45.7 (16)	0	0	39.3 (35/89)
Total % (*n*)	22.2 (90)	28.9 (117)	37.0 (150)	8.4 (34)	3.5 (14)	27.3 (405/1,486)

In the univariable predictor analysis, age at treatment start (HR 0.99, 95% CI 0.97–0.99, *p* < 0.05), EASI score at 6 months (HR 1.07, 95% CI 1.03–1.12, *p* < 0.05), DLQI score at 6 months (HR 1.12, 95% CI 1.07–1.17, *p* < 0.05), and itch-NRS at 6 months (HR 1.29, 95% CI 1.14–1.47, *p* < 0.05) were associated with overall drug survival in patients treated with MTX. In dupilumab-treated patients, the univariable analysis showed an association with EASI score at 6 months (HR 1.05, 95% CI 1.01–1.10, *p* < 0.05), DLQI score at 6 months (HR 1.10, 95% CI 1.06–1.14, *p* < 0.05), and itch-NRS at 6 months (HR 1.26, 95% CI 1.15–1.37, *p* < 0.05). For the grouped JAK inhibitors, similar associations were observed for EASI score at 6 months (HR 1.06, 95% CI 1.01–1.11, *p* < 0.05), DLQI score at 6 months (HR 1.12, 95% CI 1.07–1.17, *p* < 0.05), and itch-NRS at 6 months (HR 1.29, 95% CI 1.10–1.50, *p* < 0.05). No significant associations between covariates and treatment with tralokinumab or CsA were seen in the univariable analysis.

In the multivariable Cox regression analysis, no statistically significant effects on HR were detected for either dupilumab, MTX, or the grouped JAK inhibitors, when the predictors significantly associated with overall drug survival in the univariable model were included as covariates.

## DISCUSSION

In the present study, we aimed to investigate the real-world efficacy of systemic treatment of AD in a Swedish context using drug survival analysis.

With a 2-year drug survival of 79.7% in patients receiving dupilumab in our cohort, this study confirms the treatment persistence shown in previous studies, with similar 2-year drug survival rates of 88% and 93.4% in Dutch and Italian cohorts, respectively (17, 18). The slightly lower drug survival shown in the present study may be attributable to new treatment options having become available since 2020 or differences in the availability of alternative treatments between the different cohorts. The discontinuation rate of dupilumab was shown to be significantly lower compared with that of MTX, a finding that was also reported in the Dutch cohort study by Spekhorst et al. (17). Lack of efficacy accounted for 28.3% of dupilumab treatment cessations in our cohort, whereas adverse events accounted for 27.3%, rates similar to those seen in the Dutch (lack of efficacy 19%, adverse events 46%) and Italian (lack of efficacy 30.6%, adverse events 41.3%) cohorts (17, 18). A limitation in our data was the fairly high proportion of treatment cessations labelled as being due to “other causes”, amounting to 27.8% and 44% in subjects treated with dupilumab and CsA, respectively.

In our cohort, multivariable Cox regression analysis did not identify predictors of the overall drug survival of dupilumab. In previous studies, based on data from the Dutch BioDay Registry, use of concomitant immunosuppressive therapy at baseline and poor treatment response were associated with shorter drug survival of dupilumab due to lack of efficacy. The use of concomitant immunosuppressive therapy at baseline, older age (≥65 years), and an Investigator Global Assessment score of very severe AD were associated with shorter drug survival due to adverse events (19). A high number of patients with missing data on predictors, as presented in Table I, may have impacted the findings in the present study. Similarly, a recent investigation of drug survival of upadacitinib in AD did not identify any patient characteristics associated with drug discontinuation in uni- or multivariable regression analyses (20). In this context, it is important to note that several indicators of effective treatment at 6 months were associated with longer drug survival in unadjusted analyses. However, in the multivariable framework, these variables were included as covariates in the same models, and their shared association with the outcome may have rendered them non-significant.

The 2-year drug survival of the conventional immunomodulators MTX and CsA in our study was similar to those seen in the Dutch Bioday Registry (45.4% and 12.2% vs 41.6% and 22.5%, respectively) (17). In line with our findings, a CsA drug survival rate of 11% after 16 months of treatment was reported in an Italian study (21). The low survival rate of CsA is expected, given its main use as a short-term rescue therapy in AD. This might also explain the high frequency of discontinuation due to “other causes” mentioned earlier.

Although a 1.5-year upadacitinib drug survival of 80.2% has been reported in an Italian retrospective cohort of 325 adult AD patients (20), the 2-year drug survival of upadacitinib in our cohort was 46.0%. The leading causes of drug discontinuation for upadacitinib in our cohort were lack of efficacy, in 45.7% of cases, and side effects, in 42.9%. The corresponding figures in the Italian cohort were adverse effects in 16 out of 25 and ineffectiveness in 2 out of 25 cases of discontinuations, respectively.

The lower drug survival seen in our cohort may be explained by differences in participating clinics. Eight out of 11 participating departments (all of which were hospital-based) in the Italian cohort were located at a university hospital, whereas the SwedAD registry includes both hospital-based and non-hospital-based dermatological clinics. The facilities and support provided at a hospital clinic may translate into a decreased tendency to terminate treatments unless such action is clearly indicated. The novelty and lack of experience of JAK inhibitors during the study period may also be a factor in the decisions by physicians and patients to discontinue treatment. Lastly, the availability of alternative treatment options may also impact the drug survival of a certain medication and lower the threshold for treatment discontinuation due to, for example, lack of efficacy or concerns regarding potential adverse effects.

The 1-year overall drug survival of tralokinumab treatment of 63.5% in our study is lower than the 85.9% 1-year drug survival reported by Pezzolo et al. in an Italian cohort of 171 patients (22). The small number of patients who had received tralokinumab treatment in our cohort (*n* = 18) may reduce the generalizability of our findings. Likewise, the small sample size of patients who had received either abrocitinib (*n* = 23) or baricitinib (*n* = 30) in our cohort needs to be taken into consideration when interpreting the results of the drug survival analyses for these interventions. Further, the small number of drug discontinuation events in several of the treatment populations had a negative impact on the possibility to identify predictors of drug survival in these groups.

Our findings are consistent with those of a recent international real-world study by Torres et al. (23), which demonstrated high drug survival for dupilumab (86.3% at two years) in patients with atopic dermatitis across multiple centres in Europe and Canada. In this international cohort, the drug survival of upadacitinib after 1 and 2 years of treatment (90.2% and 78.7%, respectively) was, however, higher than the results presented in our study.

### Strengths and limitations

Although the drug survivals of MTX, CsA, dupilumab, tralokinumab, and JAK inhibitors have been reported previously, this real-world study is – to our knowledge – the first to incorporate all of these treatments in the same analysis, and including both children and adults. Due to the lack of information regarding systemic treatments received before entering the registry, this potential predictor of drug survival – which may be assumed to be relevant to treatment outcomes – could not be included in the analysis. The impact of the evolving treatment arsenal in AD needs to be kept in mind when interpreting our results. During the period examined (2017–2024), several new therapeutic options were authorized by the European Medicines Agency (dupilumab in 2017, baricitinib in 2020, tralokinumab and upadacitinib in 2021, and abrocitinib in 2022) and subsequently made available for use in Sweden (24). As not all treatment options were available during the initial years of the examined time period, the drug discontinuation rate of the treatments that were available, such as dupilumab, may be assumed to have been lower during these initial years, affecting the global drug survival of those treatments in our cohort. To ensure a fair comparison between treatment options, future studies examining the drug survival of these agents in an environment in which they are equally available is warranted. To summarize, the main limitations of the present study are the lower number of treatment episodes as well as shorter follow-up periods for some of the investigated therapies, primarily substances that have been available during a limited period of time.

### Conclusion

The drug survival data in this prospective cohort of Swedish AD patients supports previous reports of a good survival rate of dupilumab in real-world settings. This was our main finding. Further, we found that the drug discontinuation rate of dupilumab was significantly lower than that of MTX. Discrepancies in the availability of the JAK inhibitors and tralokinumab compared with those of CsA, dupilumab, and MTX during the investigated time period need to be taken into consideration when interpreting the results. Future studies of long-term prospective drug survival allowing for comparison between all available treatment options are needed.
